# Supplementary material for: Light chain mutations contribute to defining the fibril morphology in systemic AL amyloidosis
Source: Nat Commun. 2024 Jun 15;15:5121. doi: 10.1038/s41467-024-49520-6 (PMC11180120; doi:10.1038/s41467-024-49520-6)
Supplement: Supplementary file 1 — Supplementary Information [file 41467_2024_49520_MOESM1_ESM.pdf]

Supplementary Information

Light chain mutations contribute to defining the fibril morphology in systemic AL amyloidosis

Sara Karimi-Farsijani, Peter Benedikt Pfeiffer, Sambhasan Banerjee,

Julian Baur, Lukas Kuhn, Niklas Kupfer, Ute Hegenbart, Stefan O. Schönland,

Sebastian Wiese, Christian Haupt, Matthias Schmidt, Marcus Fändrich

Supplementary Methods

Fibril extraction from FOR010 heart tissue

Fibril extraction was performed according to a previously described fibril extraction protocol[1]. In short, a 250 mg piece of heart tissue from patient FOR010 was minced using a scalpel and placed in 0.5 mL of ice-cold Tris calcium buffer. The sample was homogenised using a pellet pestle and centrifuged for 5 min at 3,100 × g and 4 °C. The supernatant was removed and the washing and centrifugation steps were repeated four more times. The resulting pellet was resuspended in 1 mL solution of 5 mg/mL collagenase (Sigma) in Tris calcium buffer, containing EDTA-free protease inhibitor (Roche) and incubated overnight at 37 °C. Following the incubation, the sample was subject to a centrifugation step at 3,100 × g for 30 min. After removing the supernatant, the pellet was subjected to four washing and centrifugation steps in 0.5 mL Tris EDTA buffer for 5 min at 3,100 × g and 4 °C. The resulting pellet was then subjected to eight washing and centrifugation steps with 0.1 mL ice-cold water for 5 min at 3,100 × g and 4 °C. All supernatants were stored at 4 °C.

Denaturing protein gel electrophoresis

Samples for denaturing protein gel electrophoresis were prepared by mixing 6 µL of the respective protein sample with 1.2 µL 10 × NuPAGE reducing agent (Thermo Fisher Scientific), 4 µL 4 × NuPAGE LDS sample buffer (Thermo Fisher Scientific), and 0.8 µL water. The mixture was heated at 95 °C for 10 min and loaded onto a 4–12% NuPAGE Bis-Tris gel (Thermo Fisher Scientific). BlueEasy prestained protein ladder (Genetics) was loaded as a marker. The gel was then stained in a solution containing 30 % (v/v) ethanol, 10 % (v/v) acetic acid, and 0.25 % (w/v) Coomassie brilliant blue and destained with a solution containing 20 % (v/v) ethanol and 10 % (v/v) acetic acid.

Platinum side shadowing

A sample volume of 1.5 µL from the AL fibrils was applied to glow discharged formvar and carbon-coated 200 mesh copper grids (Plano) and incubated at room temperature until the solution was dried up. Platinum particles were evaporated at an angle of 30 ° onto the grid to form a 1 nm-thick layer by use of a BAF 300 coating device (Balzers). Grids were analysed using a S-5200 SEM (Hitachi) at an acceleration voltage of 30 kV.

Determination of the total mass of the FOR010 fibril protein

A sample of FOR010 amyloid fibrils in pure water was lyophilized. The dry protein was quantified gravimetrically and stored at -20 °C. The dry protein was dissolved in water at a concentration of 1 mg/mL. Fibrils were disrupted by incubating 2 µL of the sample with 4 µL 6 M guanidinium hydrochloride, 50 mM Tris, pH 8.0, overnight at 4 °C. The sample was diluted to 15 µL with 0.1 % (v/v) trifluoroacetic acid (TFA) and applied to a U3000 RSLCnano high-performance liquid chromatography (HPLC) system (Thermo Fisher Scientific). The sample was first injected on an Acclaim PepMap 100 (0.3 mm x 5 mm) pre-column (Thermo Fisher Scientific) which was equilibrated with 5 % (v/v) HPLC solvent B (0.1 % (v/v) formic acid (FA), 86 % (v/v) acetonitrile (ACN)). Salts and other polar contaminants were removed by washing with HPLC solvent A (0.1 % (v/v) FA) for 5 min at a flow rate of 30 µL/min. Subsequently, proteins were eluted onto an Acclaim PepMap RSLC nanoViper (0.075 mm x 500 mm) analytical column (Thermo Fisher Scientific) by changing the flow path and applying a linear gradient of 5 % to 40 % HPLC solvent B at a flow rate of 250 nL/min over 30 min. Elution of the sample from the analytical column was carried out by applying the same gradient used for the pre-column.

Eluting fractions were directly injected into the electrospray module of an LTQ Orbitrap Elite mass spectrometer (Thermo Fisher Scientific). Ionization was performed using a

nanoelectrospray ion source with distal-coated SilicaTips (New Objective). The mass spectrometer was operated with Thermo Xcalibur 2.2 SP1.48 39 software (Thermo Fisher Scientific) and the following parameters were set to acquire the mass spectra: spray voltage: 1.5 kV, capillary temperature: 250 °C, S-Lens radio frequency level: 68.9 %. Mass spectra were acquired in the Orbitrap at a resolution of 30,000 with automatic gain control enabled (106 ions; maximum fill time: 500 ms). The resulting m/z scans ranged from 370 to 1,700. Calibration of the mass spectrometer was performed using Pierce LTQ ESI Positive Ion Calibration Solution (Thermo Fisher Scientific). The recorded mass spectra were summed using Thermo Xcalibur 3.0.63 (Thermo Fisher Scientific) and deconvoluted using the "Quick deconvolution" function of MASH Explorer[2] (default settings). Monoisotopic masses of deconvolutions with a confidence score of 90 % or higher corresponding to proteins with 5 or more charges were used for the subsequent sequence assignment.

FOR010 Fibril protein sequencing

The lyophilized protein was dissolved in water to reach a concentration of 1 mg/mL. The sample was applied onto five wells of a 4–12% NuPAGE Bis-Tris gel (Thermo Fisher Scientific). The fibril protein bands were cut out and each gel slice was first washed with 0.1 mL protease buffer for 10 min. Afterwards, gel slices were washed with a 1:1 mixture of protease buffer and ACN for 10 min. The following protease buffers were used: trypsin: 50 mM $(NH_4)HCO_3$, pH 8.0; LysC: 50 mM $(NH_4)HCO_3$, pH 8.0; chymotrypsin: 50 mM Tris, pH 8.0; elastase: 50 mM Tris, 10 mM $CaCl_2$, pH 9.0; pepsin: 40 mM HCl, pH 1.5. After repeating the alternating washing steps two more times, the gel pieces were vacuum dried using a vacuum chamber, reduced in dithiothreitol (DTT) buffer (50 mM $(NH_4)HCO_3$, 55 mM DTT, pH 8.0) for 20 min at room temperature and alkylated in alkylation buffer (50 mM $(NH_4)HCO_3$, 5 mM iodoacetamide, pH 8.0) for 20 min at 37 °C. Reduced and alkylated proteins in the gel were digested by incubating each gel slice in 10 µL of a protease buffer containing 0.33 ng of the respective protease

(trypsin, LysC, elastase, pepsin) overnight at 37 °C, while the gel treated with chymotrypsin was incubated overnight at 25 °C. The peptides were extracted by transferring the gel slices into 10 µL of ACN mixed with 10 µL of 0.1 % (v/v) TFA and incubating them in an ultrasonic bath for 10 min. Subsequently, the gel slices were removed, ACN was evaporated and the remaining peptides were diluted to 15 µL with 0.1 % (v/v) TFA.

HPLC purification of the peptides and operation of the mass spectrometer was performed as described for the total mass analysis (see above). Peptide fragmentation was achieved by collision induced dissociation (CID) or higher-energy C-trap dissociation (HCD). For CID, the 20 most intense ions were selected from the survey scan. The automated gain control was set to 10,000 ions with a maximum fill time of 10 ms and Fourier-transformation preview scan was enabled. The collision energy was set to 35 %, and the activation time was set to 10 s with an activation Q of 0.25. Singly charged ions were not fragmented, and m/z of fragmented ions was excluded for 60 s. Fragment detection was performed in the quadrupole at normal scan speed. For HCD fragmentation, ten ions were selected per survey scan and singly charged ions were rejected. m/z values of fragmented ions were excluded for 60 s for additional selection. The automatic gain control was set to 50,000 ions with a maximum fill time of 100 ms. The activation time was set to 100 ms, and resulting peptide fragments were detected in the Orbitrap at a resolution of 15,000. Mass spectra were recorded and de novo sequencing of the selected peptides was performed using the PEAKS AB 2.0 software[3]. Peaks AB was operated with a mass accuracy of 15 ppm on intact peptide masses and either 0.05 Da or 0.5 Da for fragmented ions. Amino acids with a confidence level of 85 % or more were considered correct. Peptide sequences were analysed for the following PTMs: the carbamidomethylation which results from alkylation of cysteine, asparagine and glutamine deamidation and methionine oxidation. Amino acids with indistinguishable masses, such as isoleucine and leucine, as well as glutamine and lysine, were assigned according to the GL sequence.

Supplementary Figures and Figure legends

SI Figure 1

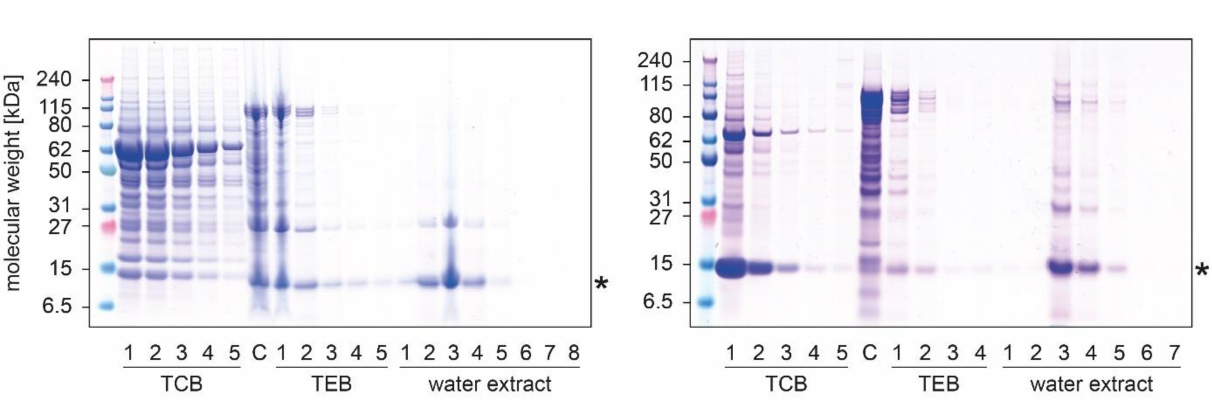

SI Fig. 1

Isolation of the FOR103 and FOR010 AL fibril proteins.

Coomassie-stained denaturing protein electrophoresis gel of fractions from the extraction of AL amyloid fibrils from the tissue. Left: FOR103; right: FOR010. Asterisk: fibril protein. TCB: supernatants of the washing steps with Tris calcium buffer; C: pellet after collagenase treatment, TEB: supernatants of the washing steps with Tris EDTA buffer; water extract: supernatants of the water extraction steps, containing the isolated amyloid fibrils. Source data are provided as a Source Data file.

SI Figure 2

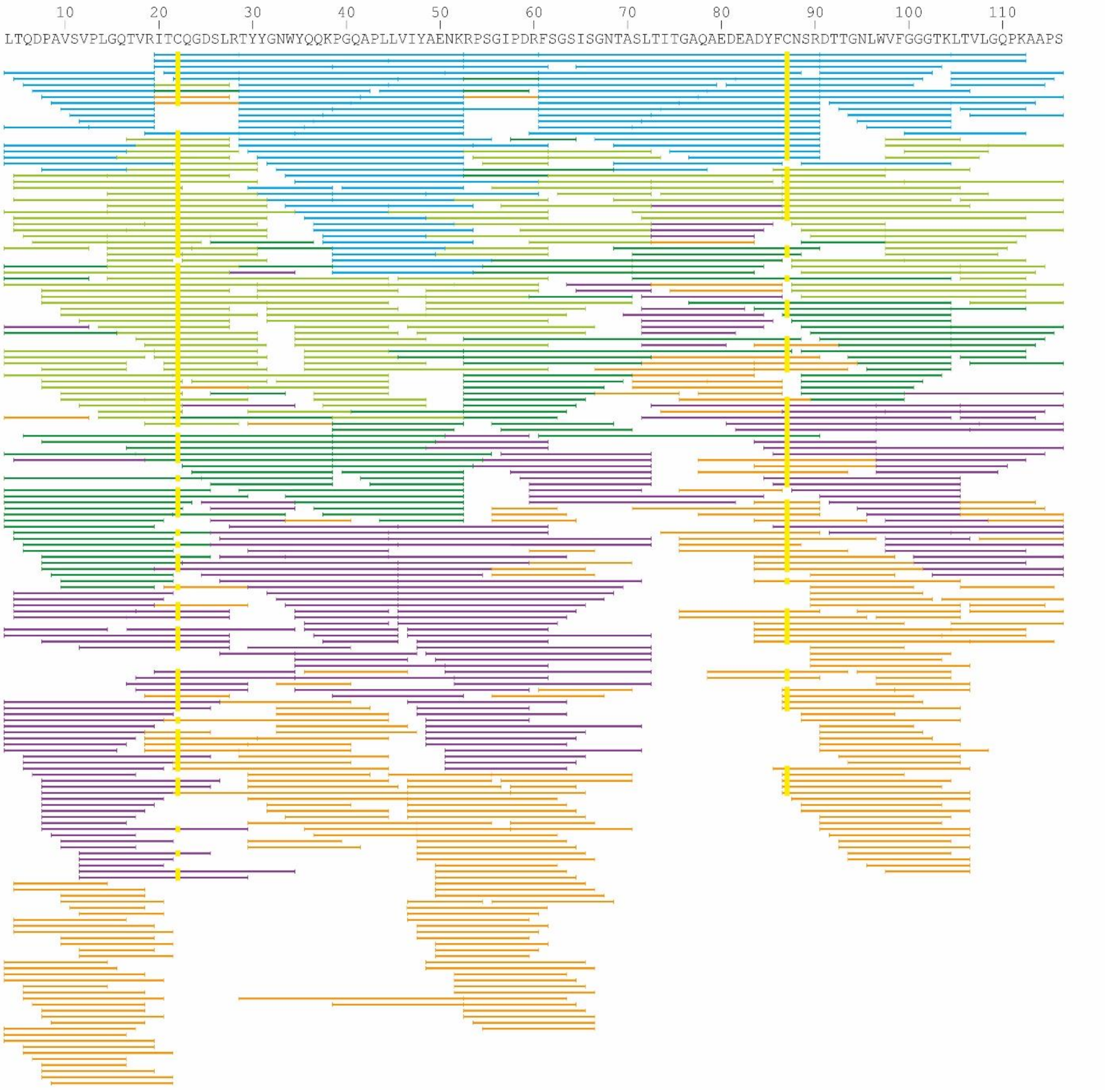

SI Fig. 2

FOR010 fibril protein fragments detected after proteolysis.

Peptide fragments detected by MS after digestion of the FOR010 fibril protein with different proteases: trypsin (blue), LysC (dark green), chymotrypsin (light green), elastase (orange) and pepsin (purple). Only a fraction of the identified peptides is shown in the panel (n = 1, biological replicate).

SI Figure 3

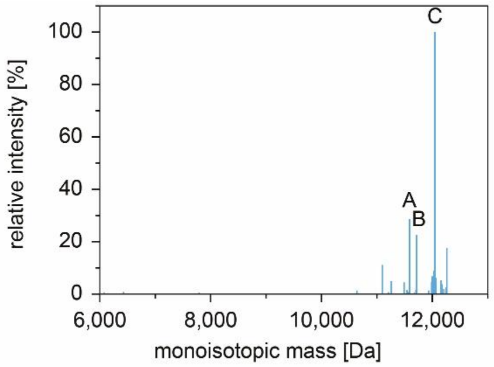

SI Fig. 3

Mass spectrum of the FOR010 fibril protein.

Mass spectrum of the undigested FOR010 fibril protein (n = 1, biological replicate). Three major peaks (A-C) are visible at 11,587.8 (A), 11,718.9 (B) and 12,045.0 Da (C). For assignments of their masses see SI Table 1.

SI Figure 4

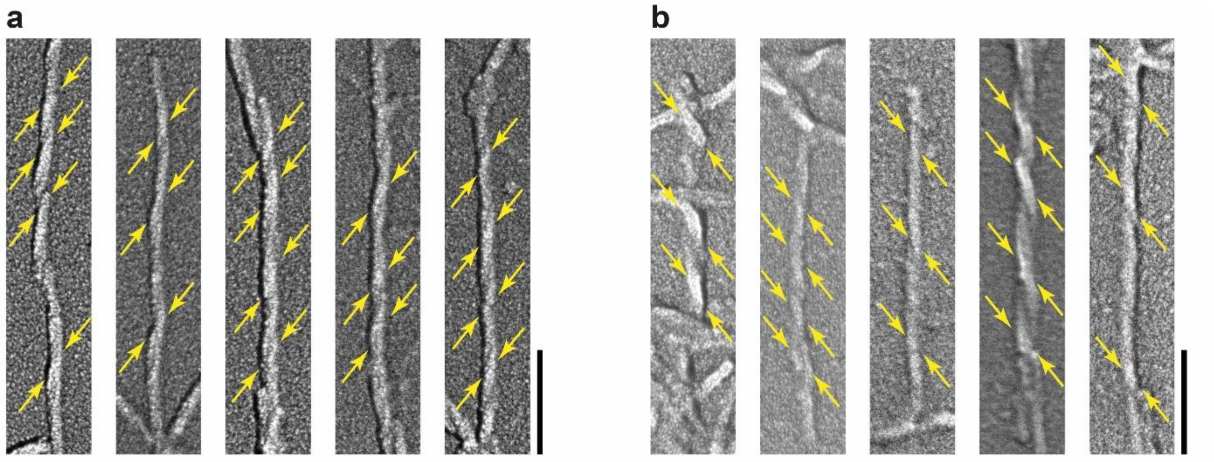

SI Fig. 4

Handedness of FOR103 and FOR010 AL amyloid fibrils.

SEM images of the AL amyloid fibrils after platinum side shadowing. (a) FOR103 fibrils are right-hand twisted. (b) FOR010 fibrils are left-hand twisted. Arrows were drawn to guide the eye. Scale bar: 100 nm.

SI Figure 5

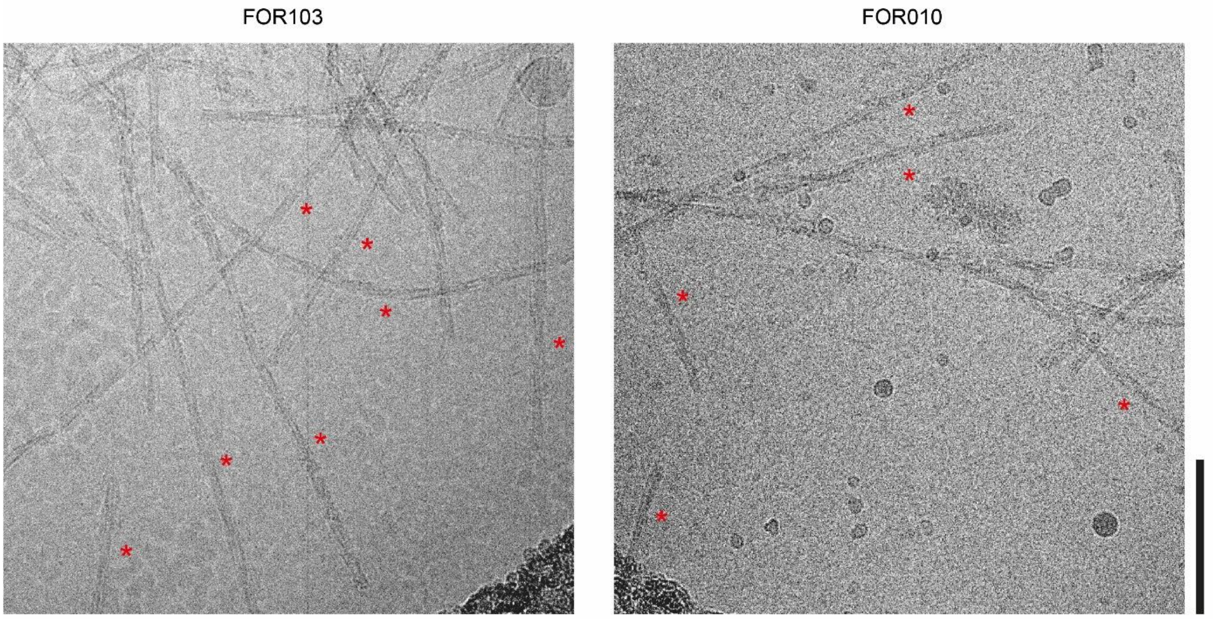

SI Fig. 5

Cryo-EM images of FOR103 and FOR010 fibrils.

Cryo-EM micrographs of FOR103 (left) and FOR010 amyloid fibrils (right). Scale bar: 100 nm. Examples of the main fibril morphology are highlighted with asterisks.

SI Figure 6

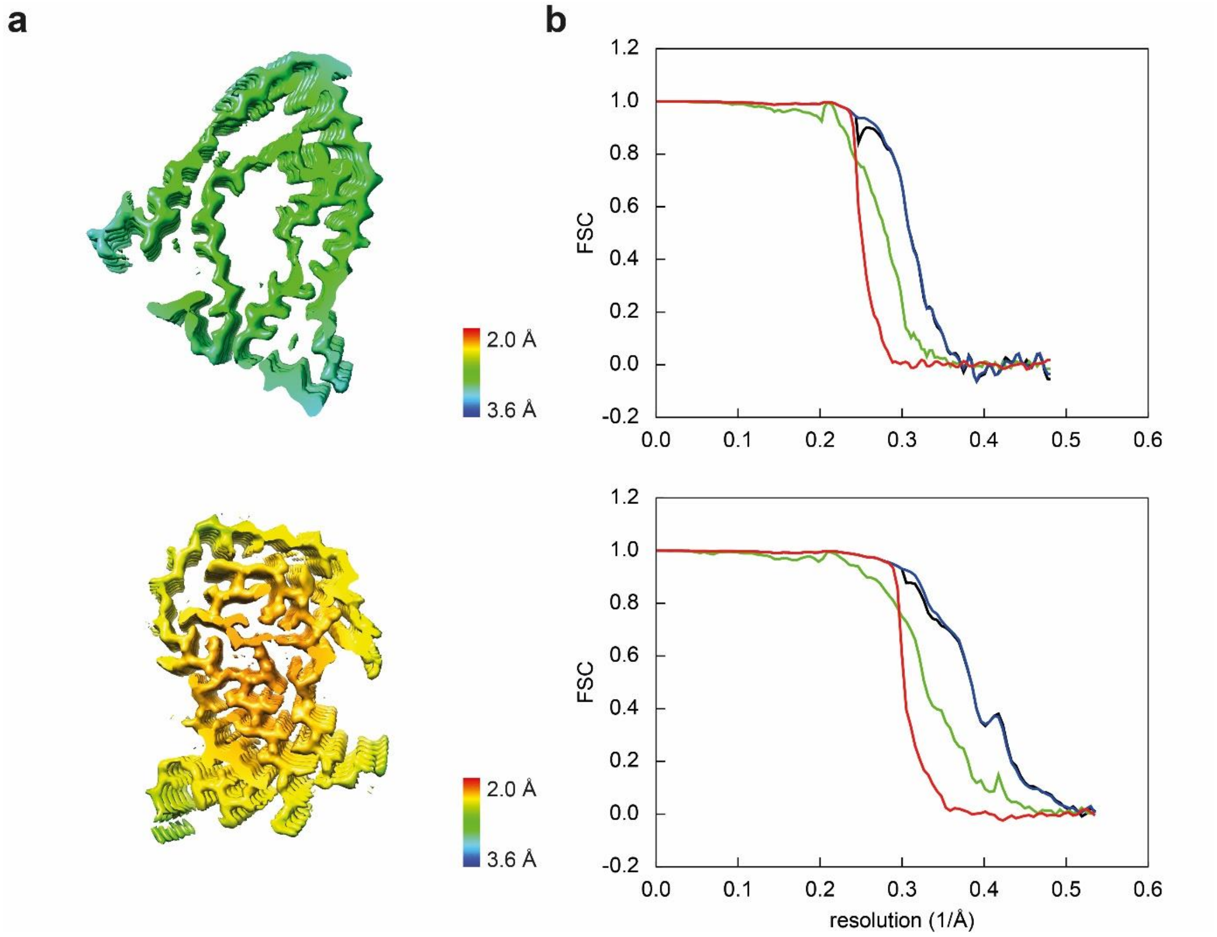

SI Fig. 6

Reconstructed 3D maps of FOR103 and FOR010 fibrils.

(a) Estimation of the local resolution of the 3D density maps. Top: FOR103; bottom: FOR010.

(b) FSC curves of the two reconstructed half maps of the two fibrils. Top: FOR103; bottom: FOR010. FSC corrected (black), FSC unmasked maps (green), FSC masked maps (blue) and FSC phase randomized masked maps (red). Source data are provided as a Source Data file.

SI Figure 7

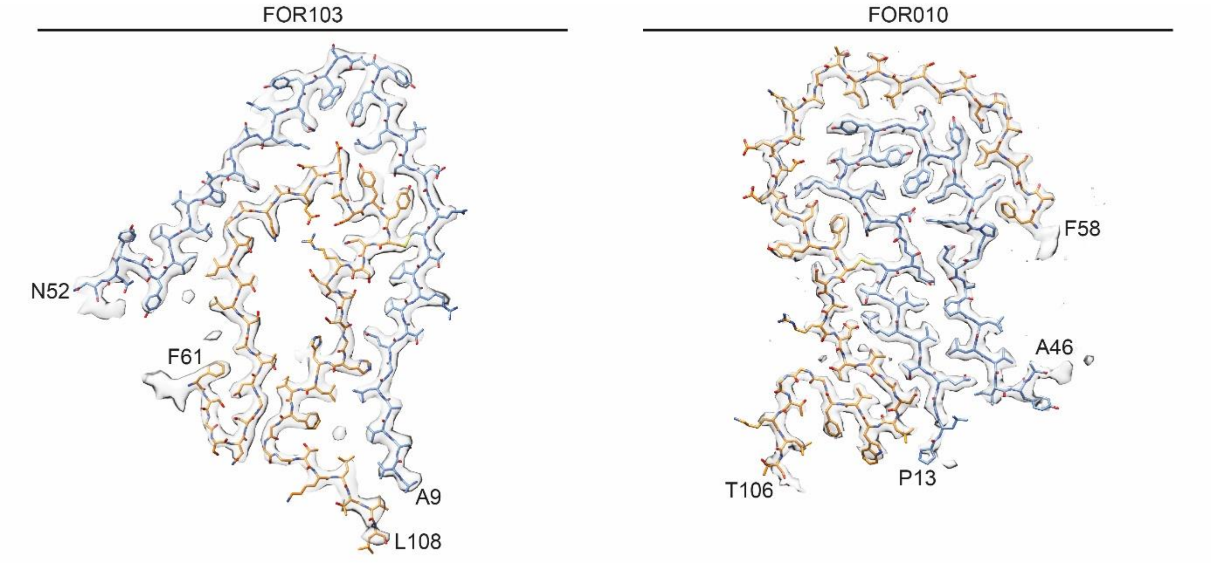

SI Fig. 7

3D maps and models of FOR103 and FOR010 fibrils.

Cross-sectional views of one molecular layer of the reconstructed 3D maps (grey) of the FOR103 (left) and the FOR010 (right) fibril, overlaid with the respective molecular model. The N-terminal ordered region is coloured in blue, while the C-terminal region is coloured orange.

SI Figure 8

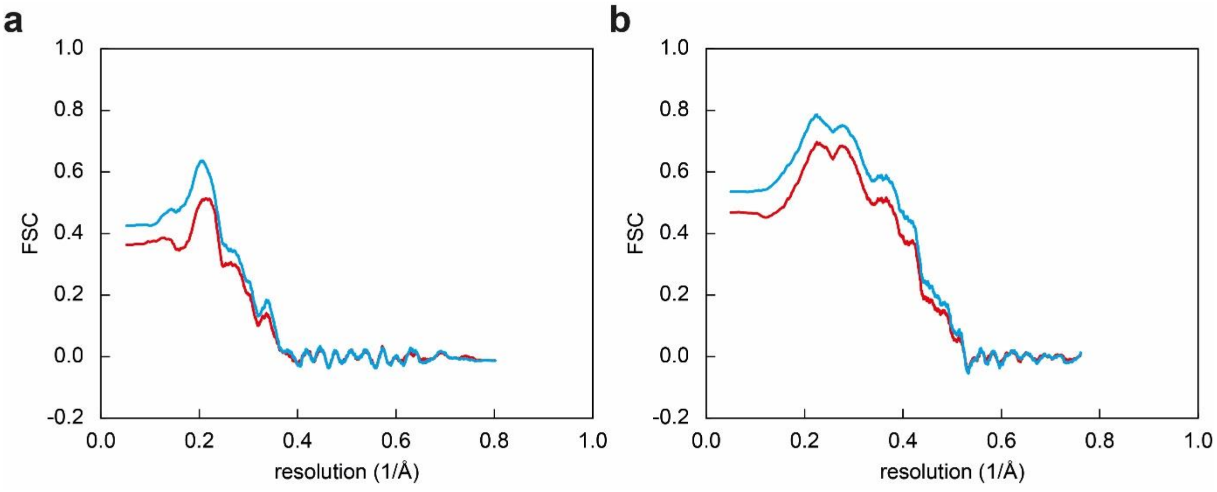

SI Fig. 8

Map-model FSC curves.

Map-model FSC curves of (a) FOR103 and (b) FOR010. Unmasked FSC (red) and masked FSC curves (blue) are shown. Source data are provided as a Source Data file.

SI Figure 9

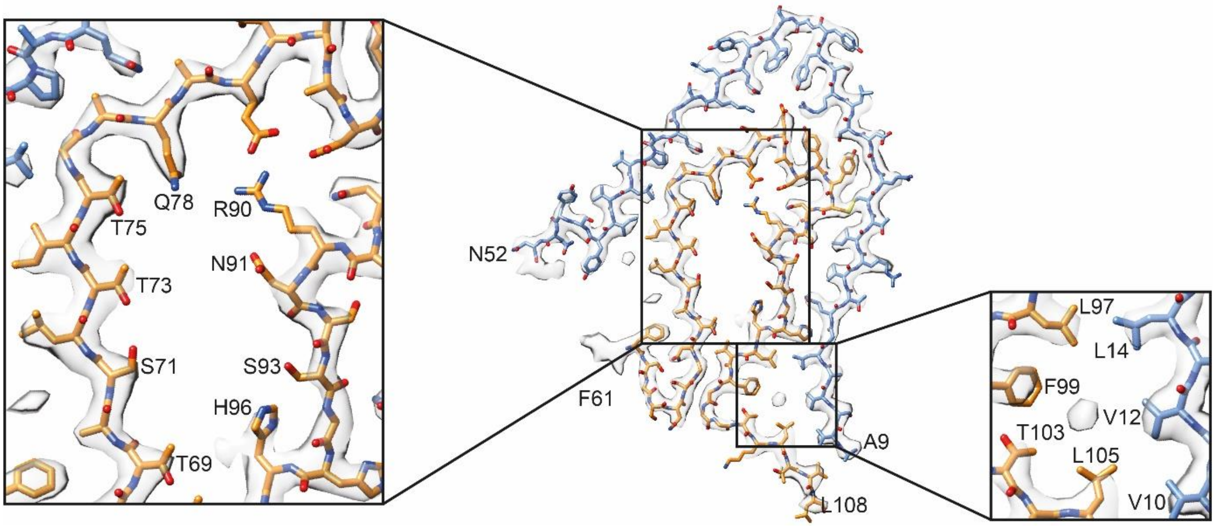

SI Fig. 9

Cavities of the FOR103 fibril protein.

Cross-sectional view of one molecular layer of the reconstructed 3D map (grey) of the FOR103 fibril, overlaid with the molecular model (sticks). The close up to the left indicates a large cavity that could be water filled. The close up to the right shows a hydrophobic cavity that is filled with density.

SI Figure 10

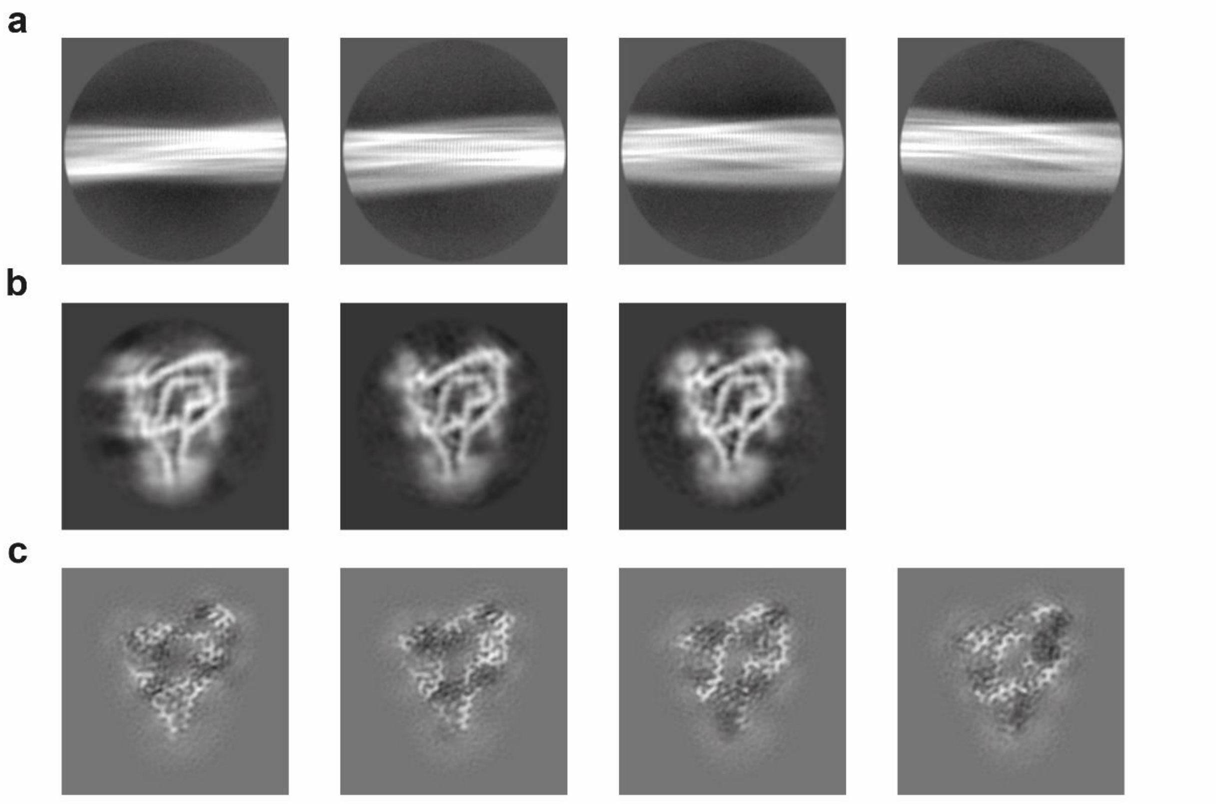

SI Fig. 10

Cryo-EM data processing of the FOR103 fibril.

(a) Representative 2D class averages of the FOR103 fibril. (b) Representative 3D classes. (c) Images of four cross-sectional sections of the reconstructed 3D map.

SI Figure 11

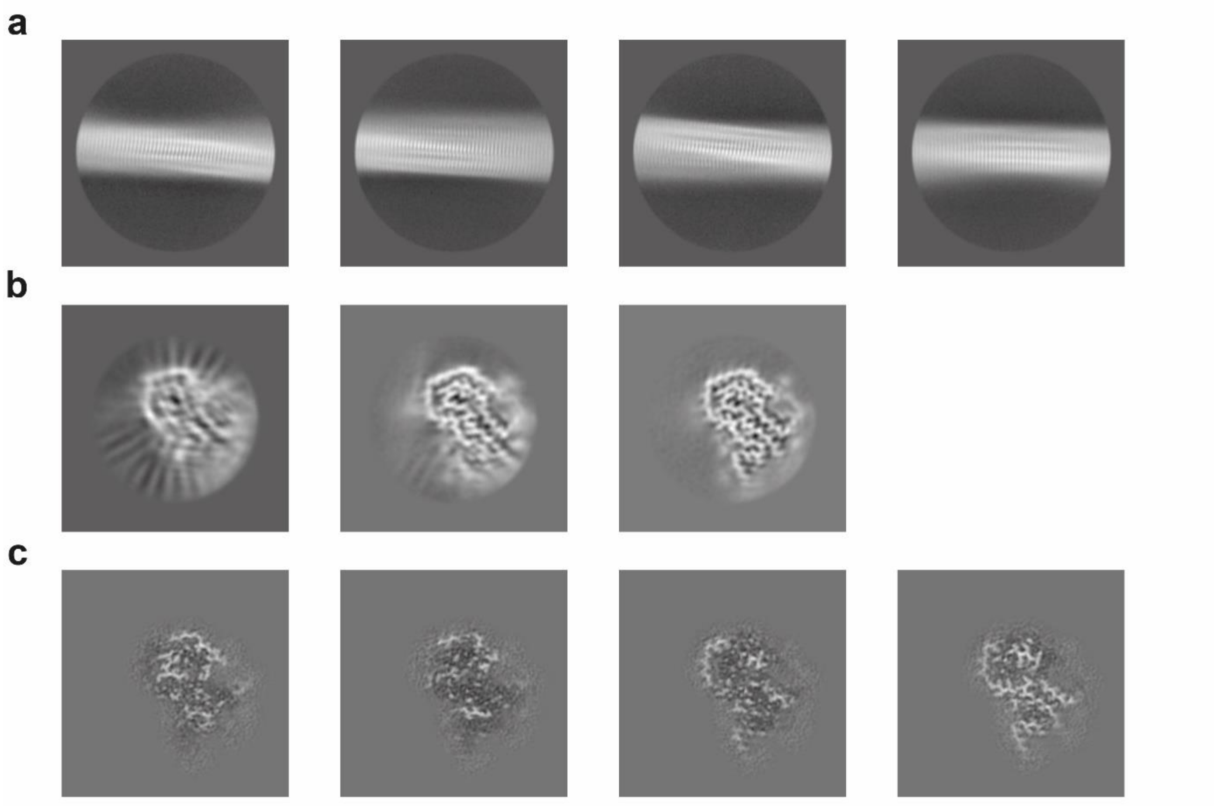

SI Fig. 11

Cryo-EM data processing of the FOR010 fibril.

(a) Representative 2D class averages of the FOR010 fibril. (b) Representative 3D classes. (c) Images of four cross-sectional sections of the reconstructed 3D map.

Supplementary Tables

SI Table 1

**Peak**	**Mass [Da]**		**Assigned LC fragment [residue - residue]**	**PTM**
	**experimental**	**theoretical**		
A	11,587.8	11,587.8	8 - 116	disulfide bond
B	11,718.9	11,718.8	4 - 112	disulfide bond
C	12,045.0	12,045.0	4 - 116	disulfide bond

SI Table 1

Assignment of the FOR010 fibril protein masses.

The assignment of the mass is provided for the three major peaks (A, B, C) of the undigested fibril protein observed by MS.

SI Table 2

	**FOR103**	**FOR010**
**Microscope**	Titan Krios (Thermo Fisher Scientific)	Titan Krios (Thermo Fisher Scientific)
**Camera**	Quantum K2	Falcon4i
**Acceleration voltage (kV)**	300	300
**Magnification**	130,000 x	130,000 x
**Defocus range (μm)**	- 1.2 to - 2.5	- 0.8 to 2.0
**Total dose (e^-/Å^2)**	53.7	40
**Number of movie frames**	40	-
**Number of EER fractions**	-	963
**Exposure time (s)**	10	3
**Pixel size (Å)**	1.04	0.934
**Mode**	Counting mode	Counting mode
**Box size (pixel)**	300	200
**Inter box distance (Å)**	33.264	30.84
**Number of extracted segments**	157,862	414,351
**Number of segments after 2D classification**	143,771	414,351
**Number of segments after 3D classification**	120,039	51,394
**Resolution, 0.143 FSC criterion (Å)**	2.92	2.25
**Map sharpening B-factor (Å^2)**	- 66.2163	- 46.3397
**Helical rise (Å)**	4.752	4.76311
**Helical twist (°)**	1.309	- 1.4634
**Imposed symmetry**	C1	C1

SI Table 2

Statistics of cryo-EM data collection and image processing.

SI Table 3

	**FOR103**	**FOR010**
**Initial model**	de novo	de novo
**Model resolution (Å)**	3.1	2.0
**Model composition**		
Non-hydrogen atoms	4,140	3,780
Protein residues	552	498
Ligands	0	0
**RMSDs**		
Bond lengths (Å)	0.014	0.012
Bond angles (Å)	2.212	2.210
**Validation**		
MolProbity score	0.5	0.5
Clash score	0.0	0.0
Poor rotamers (%)	0	0
**Ramachandran plot**		
Favoured (%)	100	100
Allowed (%)	0	0
Disallowed (%)	0	0
**EMRinger score**		
z score	9.20	10.43
score	5.02	6.28
**Map CC**		
CC mask	0.56	0.76

SI Table 3

Structural statistics of model building and refinement.

References

1. Annamalai, K. *et al.* Polymorphism of Amyloid Fibrils In Vivo. *Angew. Chem. Int. Ed.* **55**, 4822–4825 (2016).
2. Wu, Z. *et al.* MASH Explorer: A Universal Software Environment for Top-Down Proteomics. *J. Proteome Res.* **19**, 3867–3876 (2020).
3. Tran, N. H. *et al.* Complete De Novo Assembly of Monoclonal Antibody Sequences. *Sci. Rep.* **6**, 31730 (2016).
