# Supplementary material for: Light chain mutations contribute to defining the fibril morphology in systemic AL amyloidosis
Source: Nat Commun. 2024 Jun 15;15:5121. doi: 10.1038/s41467-024-49520-6 (PMC11180120; doi:10.1038/s41467-024-49520-6)
Supplement: Supplementary file 2 — Reporting Summary [file 41467_2024_49520_MOESM2_ESM.pdf]

nature portfolio

Corresponding author(s): Peter B. Pfeiffer

Last updated by author(s): Jun 6, 2024

Reporting Summary

Nature Portfolio wishes to improve the reproducibility of the work that we publish. This form provides structure for consistency and transparency in reporting. For further information on Nature Portfolio policies, see our Editorial Policies and the Editorial Policy Checklist.

Statistics

For all statistical analyses, confirm that the following items are present in the figure legend, table legend, main text, or Methods section.

n/a	Confirmed	
☐	☒	The exact sample size (*n*) for each experimental group/condition, given as a discrete number and unit of measurement
☐	☒	A statement on whether measurements were taken from distinct samples or whether the same sample was measured repeatedly
☐	☒	The statistical test(s) used AND whether they are one- or two-sided<br>*Only common tests should be described solely by name; describe more complex techniques in the Methods section.*
☒	☐	A description of all covariates tested
☒	☐	A description of any assumptions or corrections, such as tests of normality and adjustment for multiple comparisons
☐	☒	A full description of the statistical parameters including central tendency (e.g. means) or other basic estimates (e.g. regression coefficient) AND variation (e.g. standard deviation) or associated estimates of uncertainty (e.g. confidence intervals)
☐	☒	For null hypothesis testing, the test statistic (e.g. *F*, *t*, *r*) with confidence intervals, effect sizes, degrees of freedom and *P* value noted<br>*Give P values as exact values whenever suitable.*
☒	☐	For Bayesian analysis, information on the choice of priors and Markov chain Monte Carlo settings
☒	☐	For hierarchical and complex designs, identification of the appropriate level for tests and full reporting of outcomes
☒	☐	Estimates of effect sizes (e.g. Cohen's *d*, Pearson's *r*), indicating how they were calculated

Our web collection on statistics for biologists contains articles on many of the points above.

Software and code

Policy information about availability of computer code

Data collection	SerialEM
Data analysis	RELION v5.0, RELION v3.1.3, MotionCor2, IMOD, CTFFIND v4.1, Coot v0.9.1, Phenix v1.20.1-4487, ImageJ v1.53, Molprobity, MASH Explorer, PEAKS AB 2.0

For manuscripts utilizing custom algorithms or software that are central to the research but not yet described in published literature, software must be made available to editors and reviewers. We strongly encourage code deposition in a community repository (e.g. GitHub). See the Nature Portfolio guidelines for submitting code & software for further information.

Data

Policy information about availability of data

All manuscripts must include a data availability statement. This statement should provide the following information, where applicable:

- Accession codes, unique identifiers, or web links for publicly available datasets
- A description of any restrictions on data availability
- For clinical datasets or third party data, please ensure that the statement adheres to our policy

The cryo-EM images were deposited in the Electron Microscopy Public Image Achieve with the accession codes EMPIAR-11801 [https://www.ebi.ac.uk/empiar/EMPIAR-11801/] (FOR103) and EMPIAR-11802 [https://www.ebi.ac.uk/empiar/EMPIAR-11802/] (FOR010). The reconstructed 3D maps were deposited in the Electron Microscopy Data Bank with the accession codes EMD-19818 [https://www.ebi.ac.uk/emdb/EMD-19818] (FOR103) and EMD-18881 [https://www.ebi.ac.uk/

emdb/EMD-18881] (FOR010). The coordinate files of the fibril models were deposited in the Protein Data Bank with the accession codes 9EME [https://doi.org/10.2210/pdb9EME/pdb] (FOR103) and 8R47 [https://doi.org/10.2210/pdb8R47/pdb] (FOR010). The models of the previously published structures of FOR005 were deposited in the Protein Data Bank with the accession codes 6Z1O [https://doi.org/10.2210/pdb6Z1O/pdb] (FOR005-A) and 6Z1I [https://doi.org/10.2210/pdb6Z1I/pdb] (FOR005-B). The source data of Figure 4 and Supplementary Figures 1, 6 and 8 are available in the source data file. All unique biological materials are available from the corresponding author upon request. However, the amount of tissue available from patients FOR010 and FOR103 is limited.

Research involving human participants, their data, or biological material

Policy information about studies with human participants or human data. See also policy information about sex, gender (identity/presentation), and sexual orientation and race, ethnicity and racism.

Reporting on sex and gender	Sex or gender are not relevant for the purpose of this study.
Reporting on race, ethnicity, or other socially relevant groupings	Race, ethnicity or socially relevant groupings are not relevant for the purpose of this study.
Population characteristics	Source of human AL amyloid fibrils; age; sex; diagnosis<br>FOR103; in her 80s; female; major cardiac involvement<br>FOR010; in his 60s; male; major cardiac involvement
Recruitment	Selected based on clinical findings (Congo red staining to prove the presence of amyloid and immunostaining with antibodies specific for lambda light chains) at biopsy.
Ethics oversight	Patient's consent was obtained prior to collection of the biopsies as approved by the Ethical Committee of the University of Heidelberg (S-123/2006). Fibril extraction and biochemical analysis was performed under a valid permission from the Ethics Committee of Ulm University (203/18).

Note that full information on the approval of the study protocol must also be provided in the manuscript.

Field-specific reporting

Please select the one below that is the best fit for your research. If you are not sure, read the appropriate sections before making your selection.

☒ Life sciences ☐ Behavioural & social sciences ☐ Ecological, evolutionary & environmental sciences

For a reference copy of the document with all sections, see nature.com/documents/nr-reporting-summary-flat.pdf

Life sciences study design

All studies must disclose on these points even when the disclosure is negative.

Sample size	2,788 cryo-EM images (FOR103) and 4,008 cryo-EM images (FOR010) were collected. These data sets were used to extract 157,862 (FOR103) and 414,351 (FOR010) particles for reconstruction. The number of particles is based on the visibility of amyloid fibrils in the data sets.
Data exclusions	37,823 (FOR103) and 362,957 (FOR010) particles were excluded during 2D and 3D classification steps. Classes with low resolution were excluded.
Replication	EM data is based on a singular sample for each patient. Replication of the helical reconstruction is therefore not possible.
Randomization	Randomization was not relevant for the purpose of this study because the data represents 2 case studies of individual patients.
Blinding	Blinding was not relevant for the purpose of this study because the data represents 2 case studies of individual patients.

Reporting for specific materials, systems and methods

We require information from authors about some types of materials, experimental systems and methods used in many studies. Here, indicate whether each material, system or method listed is relevant to your study. If you are not sure if a list item applies to your research, read the appropriate section before selecting a response.

Materials & experimental systems

n/a	Involved in the study
☒	☐ Antibodies
☒	☐ Eukaryotic cell lines
☒	☐ Palaeontology and archaeology
☒	☐ Animals and other organisms
☒	☐ Clinical data
☒	☐ Dual use research of concern
☒	☐ Plants

Methods

n/a	Involved in the study
☒	☐ ChIP-seq
☒	☐ Flow cytometry
☒	☐ MRI-based neuroimaging
